# Effect of high-flow nasal cannula therapy on adults with obstructive sleep apnea: A meta-analysis

**DOI:** 10.1097/MD.0000000000045782

**Published:** 2025-11-28

**Authors:** Shuang Bian, Jiaqi Di, Kai Shi, Dongwei Yu, Yuejuan Feng

**Affiliations:** a Department of Pulmonary and Critical Care Medicine, The Affiliated Hospital of Hangzhou Normal University, Hangzhou, China.

**Keywords:** apnea–hypopnea index, high-flow nasal cannula therapy, meta-analysis, obstructive sleep apnea

## Abstract

**Background:**

Obstructive sleep apnea (OSA) is a prevalent sleep-related breathing disorder. There has been some evidence that patients with OSA may benefit from high-flow nasal cannula (HFNC). This meta-analysis aims to assess the effect of HFNC on adults with OSA.

**Methods:**

A systematic literature search was performed using PubMed, Embase, and Cochrane Library databases before April 2024. Inclusion criteria encompassed studies involving adult OSA patients treated with HFNC, assessed by respiratory polygraphy or polysomnography.

**Results:**

A total of 12 studies, comprising 327 patients, were included in the meta-analysis. The pooled analysis revealed that HFNC therapy led to a significant reduction in apnea–hypopnea index (mean difference [MD] = −8.90; 95% confidence interval [CI] −10.47, −7.34), improved mean and lowest oxygen saturations (MD = 2.56 and 8.46, respectively), and reduced oxygen desaturation index (MD = −28.4; 95% CI −33.75, −23.06). No significant changes were observed in REM sleep percentage and sleep efficiency. HFNC also showed a tendency to decrease the total arousal index by 12.22/h (95% CI −18.40, −6.04) and the respiratory arousal index by 10.65/h (95% CI −15.65, −5.66).

**Conclusion:**

HFNC therapy is a promising alternative for managing OSA. It significantly reduces apnea–hypopnea index, improves oxygenation, and decreases sleep disruptions in adults with OSA.

## 1. Introduction

Obstructive sleep apnea (OSA) is a prevalent sleep-related breathing disorder. It is marked by recurring episodes of upper airway closure during sleep, leading to intermittent hypoxemia, autonomic fluctuations, and sleep fragmentation.^[1]^ It may present as snoring, observed apneas, nocturnal gasping, sleep disturbances, daytime sleepiness, loss of memory, decline in cognitive abilities, and general fatigue. The etiology, symptoms, and consequences vary considerably between patients, resulting in under-recognition of patients with atypical symptoms.^[2,3]^ Epidemiological data revealed that the prevalence of OSA in middle-aged and older men and women was 34% and 17%, respectively.^[4]^ Untreated OSA increases the risk for future complications such as hypertension, obesity, depression, diabetes mellitus, hypercholesterolemia, coronary heart disease, heart failure, stroke, and even sudden death.^[5,6]^

Therapies for OSA include weight loss, behavioral modification, medication, surgical procedures, oral appliances, and continuous positive airway pressure (CPAP). CPAP works during sleep by opening the upper airway through positive pressure, thus decreasing resistance and collapse in the upper airway. Since it is both effective and noninvasive, CPAP has long been considered the treatment of choice for most OSA patients. However, its tolerability is not always optimal, thereby leading to nonadherence as a significant constraint.^[7,8]^ In such cases, there is a need for an alternative treatment option to manage this condition.

High-flow nasal cannula (HFNC) is a novel respiratory support technique that delivers controlled concentrations of warmed and humidified oxygen at high-flow rates through the nasal route.^[9]^ In recent times, the use of HFNC has expanded quickly in various medical settings for treating respiratory conditions like acute respiratory failure, chronic obstructive pulmonary disease, preoxygenation, apneic oxygenation for intubation, preventing reintubation, and postoperative recovery.^[10]^ HFNC can enhance oxygenation, decrease airway resistance, and offer moistened airflow to improve the openness of the pharyngeal airway in patients with OSA. Theoretically, HFNC has the potential to be considered for treating OSA as it can increase airway pressure, which may help to overcome the critical pressure in the pharyngeal airway.^[10,11]^ Compared with CPAP, HFNC offers a less intrusive interface, reduced noise, and improved humidification, which may enhance patient comfort and adherence. While CPAP remains the gold standard, HFNC emerges as a promising alternative for intolerance cases. Several studies have assessed the efficacy of HFNC for the treatment of OSA but have been limited by small sample sizes or other methodological limitations. In this meta-analysis, we investigate the therapeutic effects of HFNC for the treatment of OSA. This may provide further insights into the management of OSA.

## 2. Methods

Methods and reporting were based on Preferred Reporting Items for Systematic Reviews and Meta-Analyses guidelines. PROSPERO was used to register the research protocol (https://www.crd.york.ac.uk/prospero; CRD42024549100).

### 2.1. Literature search

A literature search of electronic databases including PubMed, Cochrane Library, and Embase was conducted up to April 2024. The disease and intervention terms were adjusted based on the index terms used in each database, including Medical Subject Headings and Emtree. There were no limitations based on language or publication date. Detailed information about the search strategy used across all databases is available in the Supplementary Materials (Table S1, Supplemental Digital Content, https://links.lww.com/MD/Q648). To identify additional eligible studies, we also reviewed the references of all included studies and published review articles.

### 2.2. Study selection

Inclusion criteria for studies included in this review were as follows: All types of studies, whether retrospective or prospective, conducted on adult patients (aged 18 years or older) with OSA. HFNC was used as a treatment for the management of OSA. Outcome data were acquired using respiratory polygraphy or polysomnography. Studies were required to include recordings at baseline and after HFNC treatment.

The exclusion criteria were as follows: Duplicate publications or overlapping datasets from the same cohort (only the latest publication with the most complete or updated data was selected). Missing data for predefined critical outcomes (apnea–hypopnea index [AHI], nocturnal oxygen saturation, or sleep architecture). Incomplete specification of the intervention protocol (e.g., unreported HFNC flow rates).

### 2.3. Quality evaluation

The quality of the included studies was evaluated by the National Institute of Health quality assessment tool for before–after (pre–post) studies with no control group (https://www.nhlbi.nih.gov/health-topics/study-quality-assessment-tools). This 12-question tool provides reviewers with an objective method for assessing a study’s internal validity. Each question can be answered as yes, no, not applicable, or not reported, and the number of affirmative answers to each question was recorded.

### 2.4. Data extraction

The literature search, studies selection, and data extraction were performed by 2 independent reviewers (SB and JD). Any discrepancies were addressed through discussion or by consulting another experienced investigator. Data extracted from studies that were retrieved are as follows: first author, publication year, location, study design, number of participants, demographic data, evaluation of study bias, HFNC flow rate, and outcomes.

### 2.5. Statistical analysis

Statistical analysis of this study was conducted with the Cochrane systematic review software Review Manager (RevMan; Version 5.4; Copenhagen: The Nordic Cochrane Centre, The Cochrane Collaboration, 2020). For continuous variables, means and standard deviations are presented, and for dichotomous variables, frequencies or percentages are given. An assessment of heterogeneity among studies was conducted using the *I*^2^ statistic (where *I*^2^ > 50% indicates high heterogeneity) as well as the Cochran *Q* test (where *P* < .05 represents heterogeneity). Fixed-effects models were applied if heterogeneity was present; random-effects models applied otherwise. Additionally, the effect of each individual study was also assessed through sensitivity analysis by sequentially excluding each study. A mean difference (MD) or standard MD with 95% confidence interval (CI) was calculated for continuous data; a risk ratio with 95% CI was calculated for dichotomous data. Funnel plots for potential publication bias were conducted. Statistical significance was determined by a 2-sided *P*-value of <.05.

## 3. Results

### 3.1. Literature search and study characteristics

**Figure 1.**
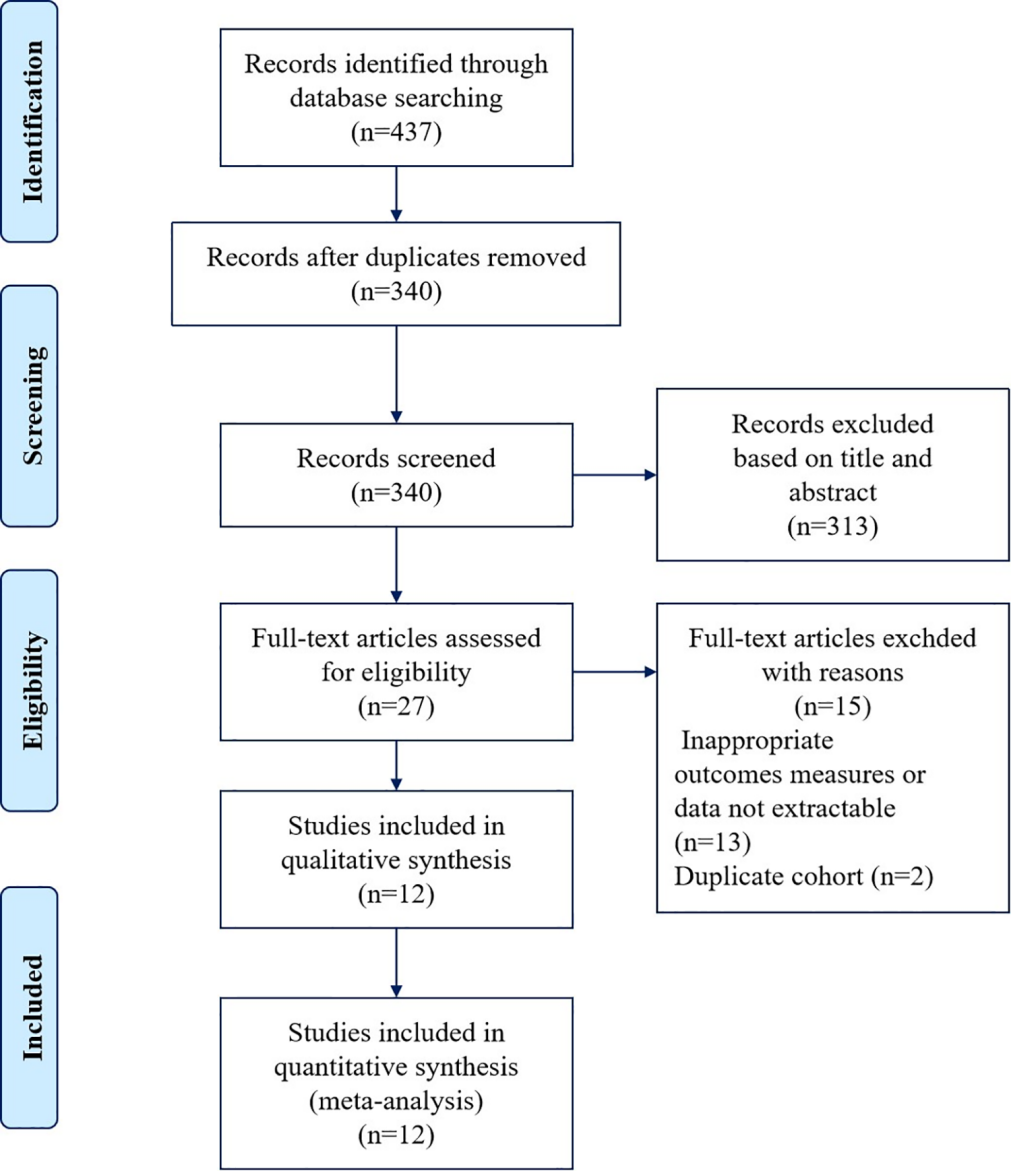
PRISMA flow diagram. PRISMA = Preferred Reporting Items for Systematic Reviews and Meta-Analyses

A total of 437 references were found during the search of the literature. After analyzing titles and authors, we removed 97 duplicates and excluded 313 studies that did not meet the criteria for inclusion. We tried to obtain the complete texts of the remaining 27 research papers. Finally, the analysis included 12 studies with 327 participants. The flowchart of the study is presented in Figure 1. The characteristics of the included studies are summarized in Table 1.

**Table 1:** Characteristics of the participants.

Authors (yr)	Location	Patient population	Study design	Comorbidities	N	Age (yr)	Male/female (n)	BMI (kg/m^2^)	Flow rate for HFNC	Monitoring device	Baseline AHI (events/h)
Li 2024	China	Stroke unit	RCT	Ischemic stroke	60	61.8 ± 11.12	40/20	24.15 ± 3.66	20–60 L/min	Standard PSG	15.5 ± 6.5
Spicuzza 2022	Italy	Sleep clinic	PC	COPD	40	75.6 ± 7.0	22/18	29.3 ± 5.2	30–60 L/min	Standard PSG	25.4 ± 8.6
Yuichi 2022	Japan	Surgical patients	Randomized crossover	Postoperative	19	65.0 ± 11.8	13/6	26.3 ± 4.6	Started at 20 L/min	Portable monitoring	59.6 ± 12.0
Tsai 2022	Singapore	Surgical patients	RCT	Postoperative	HFNC (30); CPAP (10)	49.0 ± 16.0:46.3 ± 16.7	17/13:4/6	43 ± 5.6:43.03 ± 7.1	20 L/min; 30 L/min; 40 L/min	NR	50.0 ± 35.6
Yan 2021	China	Sleep clinic	PC	None	56	45.6 ± 12.5	47/9	26.7 ± 3.1	20 L/min	Standard PSG	26.9 ± 14.7
Yu 2021	China	Sleep clinic	Randomized crossover	None	28	53.5 ± 13.7	21/7	27.4 ± 5.0	Median (IQR): 60 (40–60) L/min	Standard PSG	34.90 ± 27.7
Ho 2020	China	Stroke unit	PC	Ischemic stroke	11	72 ± 11.1	8/3	23.5 ± 3.4	50 L/min; 60 L/min	Standard PSG	52.0 ± 23.7
Nobuto 2020	Japan	Stroke unit	PC	Acute stroke	8	68 ± 12	7/1	25 ± 4.6	40 L/min	Watch PAT	21.3 ± 14
Sowho 2015	Australia	NR	Randomized crossover	None	10	51.3 ± 9.6	3/7	32.2 ± 7.7	10–35 L/min	Standard PSG	10.9 ± 5.8
Haba-Rubio 2012	Switzerland	Stroke unit	PC	Acute stroke patients	10	56.8 ± 10.7	10/0	NR	18 L/min	Standard PSG	40.4 ± 25.7
Nilius 2010	USA	Sleep clinic	PC	None	54	50.5 ± 10.4	42/12	28.0 ± 3.5	20 L/min	Standard PSG	NR
McGinley 2007	USA	Sleep clinic	Randomized crossover	None	11	49.7 ± 16.6	6/5	30.5 ± 14.3	20 L/min	Standard PSG	28 ± 16.6

CPAP = continuous positive airway pressure, HFNC = high-flow nasal cannula, NR = not reported, PC = prospective cohort, PSG = polysomnography, RCT = randomized controlled trial.

### 3.2. Bias risk of the included studies

The results of the quality assessment tool for before–after (pre–post) studies with no control group are presented in Table S1, Supplemental Digital Content, https://links.lww.com/MD/Q648 of supplemental material. Most studies met criteria for 5 to 7 of the 12 items in the quality assessment tool. The primary limitation is the small sample size across the majority of studies (Table S2, Supplemental Digital Content, https://links.lww.com/MD/Q648).

### 3.3. Meta-analysis results

#### 3.3.1. Apnea–hypopnea index

**Figure 2.**
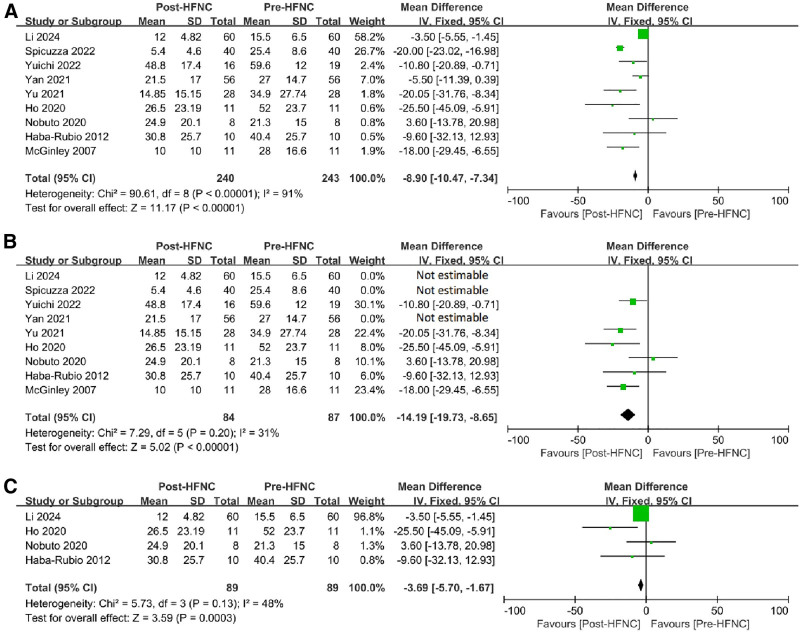
(A) Forest plot of the effect of HFNC therapy on AHI. (B) Forest plot of the effect of HFNC therapy on AHI (removing studies for heterogeneity). (C) Forest plot of the effect of HFNC therapy on AHI in stroke patients. AHI = apnea–hypopnea index, HFNC = high-flow nasal cannula.

The mean ± standard deviation (M ± SD) of AHI before and after HFNC decreased from 28.90 ± 19.77/h to 17.69 ± 17.37/h, showing a MD of −8.90 (95% CI −10.47, −7.34), Z score of 11.17 (*P* < .00001) (Fig. 2). Both the *I*^2^ statistic (91%) and the *Q* statistic (*P* < .00001) indicated significant heterogeneity. Consequently, studies were systematically excluded to pinpoint the source. After excluding Li et al, Spicuzza et al, and Yan et al, there was no heterogeneity in the remaining patients, with an *I*^2^ statistic of 31% and a *Q* statistic of 0.2. The mean difference for the remaining studies was −14.19 (95% CI −19.73, −8.65). No evidence of publication bias was found, as the funnel plot demonstrated symmetry (Figure S1, Supplemental Digital Content, https://links.lww.com/MD/Q648).

Next, we analyzed the change in AHI among stroke patients with OSA. Eighty-nine patients were included in the study. After HFNC therapy, AHI decreased by 3.69/h (95% CI −5.70, −1.67), *Z* score of 3.59 (*P* = .0003) (Fig. 2).

#### 3.3.2. Mean oxygen saturation

**Figure 3.**
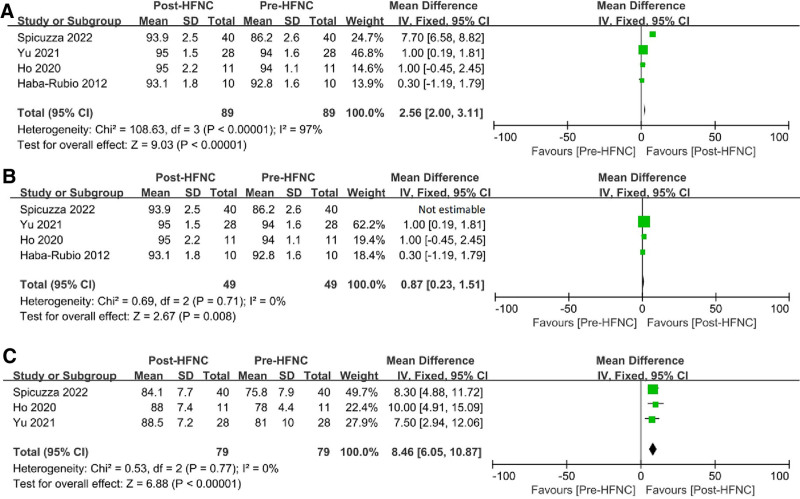
(A) Forest plot of the effect of HFNC therapy on mean oxygen saturation. (B) Forest plot of the effect of HFNC therapy on mean oxygen saturation (removing studies for heterogeneity). (C) Forest plot of the effect of HFNC therapy on lowest oxygen saturation. HFNC = high-flow nasal cannula.

There was an improvement in mean oxygen saturation from 90.36 ± 4.31% to 94.29 ± 2.19% in 89 patients. The MD was 2.56 (95% CI 2.00, 3.11), *Z* score of 9.03 (*P* < .00001). Heterogeneity was indicated by both the *I*^2^ statistic (97%) and *Q* statistics (*P* < .00001). A removal of the study by Spicuzza et al resulted in no heterogeneity (*I*^2^ = 0%, *Q* statistic value of 0.71) and an increase of 0.87 percent (95% CI 0.23, 1.51)% (Z = 2.67, *P* = .008) in mean oxygen saturation of all remaining 49 patients. This has been shown in Figure 3.

#### 3.3.3. Lowest oxygen saturation

Three studies reported the lowest oxygen saturation. The pooled analysis showed that the lowest oxygen saturation of 79 patients increased significantly from the baseline value of 77.95 ± 8.60% to 86.20 ± 7.69% after HFNC therapy. The MD was 8.46 (95% CI 6.05, 10.87), Z score of 6.88 (*P* < .00001) (Fig. 3).

#### 3.3.4. Oxygen desaturation index

Oxygen desaturation index changes were evaluated by 5 studies and demonstrated a reduction from 34.02 ± 27.65 to 11.68 ± 16.88, pre- and post-HFNC, respectively. Heterogeneity was indicated by both the *I*^2^ statistic (81%) and *Q* statistics (value of = 0.0004). After excluding the study by Nobuto et al, heterogeneity testing showed that *I*^2^ = 27%, indicating low heterogeneity. The pooled analysis showed a decrease in oxygen desaturation index by 28.4 events/h (95% CI −33.75, −23.06) in the remaining patients (Figure S2, Supplemental Digital Content, https://links.lww.com/MD/Q648).

#### 3.3.5. Rapid eye movement (REM) sleep (%)

**Figure 4.**
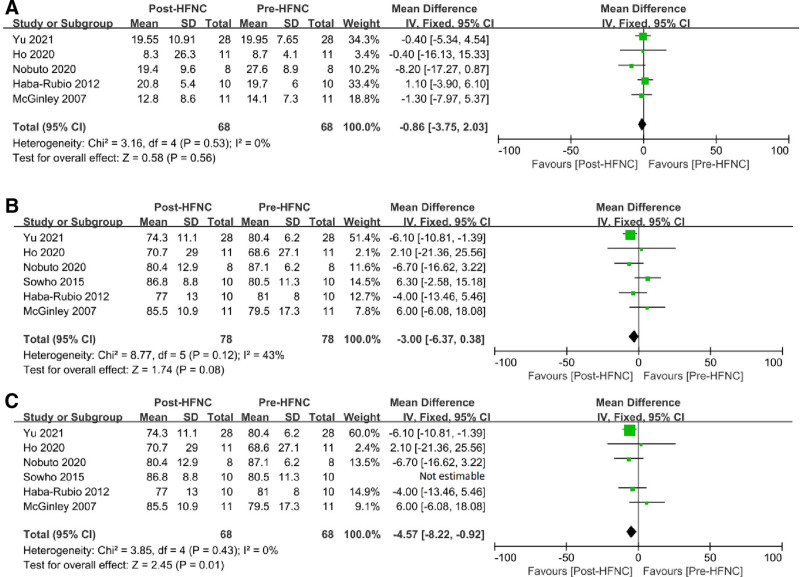
(A) Forest plot of the effect of HFNC therapy on REM sleep (%). (B) Forest plot of the effect of HFNC therapy on sleep efficiency. (C) Forest plot of the effect of HFNC therapy on sleep efficiency (removing studies for heterogeneity). HFNC = high-flow nasal cannula, REM = rapid eye movement.

With regard to sleep architecture, there were no significant differences in REM sleep (%). The pooled analysis of 5 studies revealed that patients experienced no statistically significant change in REM sleep (%), with values decreasing from 18.05 ± 8.77% at baseline to 16.80 ± 14.02% after HFNC therapy. The MD was −0.86 (95% CI −3.75, 2.03), with a *Z* score of 0.58 (*P* = .56) (*I*^2^ = 0%, *Q* statistics *P* = .53) (Fig. 4).

#### 3.3.6. Sleep efficiency

In sleep parameters, 6 studies reported sleep efficiency. There was no significant difference in sleep efficiency with significant heterogeneity (*I*^2^ = 43%, *Q* statistics *P* = .12) (Fig. 4). After excluding the study by Sowho et al to reduce heterogeneity, sleep efficiency decreased from 79.22 ± 14.47 to 76.64 ± 15.99, with a MD of −4.57 (95% CI −8.22, −0.92), *Z* score of 2.45 (*P* = .01).

#### 3.3.7. Total arousal index

There was a tendency for HFNC therapy to reduce sleep disruption markers. Total arousal index changes were evaluated by 4 studies; *I*^2^ (54%) and *Q* statistics (*P* = .09) demonstrated significant heterogeneity. With Sowho et al removed from the analysis, the pooled analysis showed a decrease in total arousal index of 12.22/h (95% CI −18.40, −6.04) (Figure S3, Supplemental Digital Content, https://links.lww.com/MD/Q648).

#### 3.3.8. Respiratory arousal index

Similarly, significant heterogeneity was observed in the pooled analysis of the respiratory arousal index (*I*^2^ = 58%, *Q* statistics of *P* = .07). After excluding the study by Ho et al to reduce heterogeneity, the pooled analysis showed a decrease in respiratory arousal index by 10.65/h (95% CI −15.65, −5.66) in the remaining patients (Figure S3, Supplemental Digital Content, https://links.lww.com/MD/Q648).

#### 3.3.9. Preference for device and comfort

In a study, CPAP was found to reduce respiratory events and improve sleep quality, as well as being more comfortable for patients than HFNC.^[12]^ Another study reported that HFNC was more comfortable than CPAP, while a third study noted similar adherence to both HFNC and CPAP over a short period of use.^[13,14]^ There are several reasons why users prefer HFNC devices, including comfort, ease of use, decreased noise levels, and a positive perception of efficacy.

For HFNC treatment, 4 studies^[11,15–17]^ reported discomfort or intolerance. There were no reports of significant discomfort or side effects in 2 studies.^[18,19]^

## 4. Discussion

In the treatment of OSA, continuous positive airway pressure (CPAP) is now recommended. Its application, however, is limited by patients’ unsatisfactory tolerance. It has been reported that between 20% and 50% of patients adhere to CPAP therapy with 4.4 hours of use per night on average. There are various reasons for this, including a difficult patient–mask interface, the inability to tolerate pressure, skin breakdowns, and claustrophobia.^[20,21]^ Recent studies have suggested that HFNC may be an alternative treatment for OSA.^[22]^ By supplying heated, humidified air at high-flow rates, HFNC can potentially reduce obstructions in the upper airways. This therapy offers a less intrusive method compared to conventional treatments, potentially improving patient compliance and comfort. A systematic review and meta-analysis of pediatric patients with OSA revealed that HFNC therapy improves obstructive AHI and minimum oxygen saturation, indicating its potential efficacy in this population.^[23]^ In adult patients, HFNC has proven to be effective in managing respiratory failure, including both hypoxemic and hypercapnic conditions.^[24]^ These studies collectively provide evidence of the potential therapeutic effects of HFNC for the treatment of OSA.

This meta-analysis included 327 adult patients from 12 studies to evaluate the efficacy of HFNC in the treatment of OSA. Our findings indicate that HFNC therapy significantly reduces AHI, improves both mean and lowest oxygen saturation levels, and decreases the oxygen desaturation index in adults with OSA. HFNC therapy also demonstrated a potential to reduce indicators of sleep disruption, as evidenced by a decreased arousal index. Additionally, in this study, the absence of significant changes in REM sleep percentage and sleep efficiency post-HFNC therapy is an intriguing observation. Sleep architecture plays a critical role in maintaining physiological homeostasis. During nocturnal sleep, non-REM and REM stages alternate cyclically, with each phase regulating essential functions including respiratory control, glucose metabolism, cardiovascular stabilization, memory consolidation, and mood regulation.^[25]^ Previous research has demonstrated a trend of reduced drowsiness sleep stages alongside elevated deep sleep and REM stages during CPAP administration.^[26]^ Compared to CPAP, our data suggest HFNC therapy improves specific respiratory parameters but demonstrates limited efficacy in optimizing sleep architecture and efficiency.

Patients with acute stroke have reported decreasing cerebral blood flow velocity with CPAP, as well as swallowing impairment and increased aspiration pneumonia risks. As a consequence, the efficacy of CPAP for dysphagic stroke patients with OSA is uncertain.^[16]^ In subgroup analysis, the reduction among AHI of 89 stroke patients with OSA suggests that HFNC therapy is a promising treatment for managing OSA in this population.

The mixed findings regarding patient preference and comfort between HFNC and CPAP therapies reflect the subjective nature of treatment tolerance. While 1 study favored CPAP for its efficacy and comfort, others found HFNC more comfortable or reported similar adherence rates. More research is required to determine the best approach to OSA treatment to optimize patient compliance and treatment outcomes.

There are several limitations to this study. Firstly, the sample size was small and the number of studies in the review was limited, with an emphasis on short-term research. Secondly, the majority of current studies were retrospective in nature, with only 2 being a randomized controlled trial, and the quality of research methods varied. Additionally, only pretreatment and posttreatment values were compared, limiting the ability to draw conclusions regarding the superiority of HFNC versus CPAP. Another limitation in the study was the presence of diverse patient groups with varying degrees of comorbidities and variations, potentially impacting the generalizability and result heterogeneity. Finally, future studies should investigate the long-term cardiovascular and metabolic impacts of HFNC, as well as patient-reported outcomes (e.g., Epworth Sleepiness Scale) to fully assess its clinical benefits.

## 5. Conclusion

In conclusion, HFNC therapy is a promising alternative for managing OSA, especially in individuals who cannot tolerate or adhere to CPAP treatment. The meta-analysis suggests that HFNC therapy can be effective in reducing AHI, improving oxygenation, and potentially decreasing sleep disruptions in adults with OSA. Due to the previously noted limitations, further larger-scale studies are warranted to elucidate the long-term efficacy and compliance of HFNC therapy for OSA.

## Acknowledgments

We would like to acknowledge the contributions of all the authors of the included studies.

## Author contributions

**Conceptualization:** Shuang Bian, Yuejuan Feng.

**Data curation:** Shuang Bian, Jiaqi Di.

**Formal analysis:** Shuang Bian, Jiaqi Di.

**Investigation:** Shuang Bian, Kai Shi.

**Methodology:** Shuang Bian, Kai Shi, Dongwei Yu.

**Software:** Shuang Bian, Dongwei Yu.

**Writing – original draft:** Shuang Bian, Jiaqi Di.

**Writing – review & editing:** Yuejuan Feng.

## Supplementary Material



## References

[1] Patel SR. Obstructive sleep apnea. Ann Intern Med. 2019;171:ITC81–96. 31791057 10.7326/AITC201912030

[2] Keenan BT Kim J Singh B. Recognizable clinical subtypes of obstructive sleep apnea across international sleep centers: a cluster analysis. Sleep. 2018;41:zsx214. 29315434 10.1093/sleep/zsx214 PMC5914381

[3] Leng Y McEvoy CT Allen IE Yaffe K. Association of sleep-disordered breathing with cognitive function and risk of cognitive impairment: a systematic review and meta-analysis. JAMA Neurol. 2017;74:1237–45. 28846764 10.1001/jamaneurol.2017.2180 PMC5710301

[4] Peppard PE Young T Barnet JH Palta M Hagen EW Hla KM. Increased prevalence of sleep-disordered breathing in adults. Am J Epidemiol. 2013;177:1006–14. 23589584 10.1093/aje/kws342 PMC3639722

[5] Gottlieb DJ Punjabi NM. Diagnosis and management of obstructive sleep apnea: a review. JAMA. 2020;323:1389–400. 32286648 10.1001/jama.2020.3514

[6] Yeghiazarians Y Jneid H Tietjens JR. Obstructive sleep apnea and cardiovascular disease: a scientific statement from the American Heart Association. Circulation. 2021;144:e56–67. 34148375 10.1161/CIR.0000000000000988

[7] Lee JJ Sundar KM. Evaluation and management of adults with obstructive sleep apnea syndrome. Lung. 2021;199:87–101. 33713177 10.1007/s00408-021-00426-w

[8] Veasey SC Rosen IM. Obstructive sleep apnea in adults. N Engl J Med. 2019;380:1442–9. 30970189 10.1056/NEJMcp1816152

[9] Rochwerg B Einav S Chaudhuri D. The role for high flow nasal cannula as a respiratory support strategy in adults: a clinical practice guideline. Intensive Care Med. 2020;46:2226–37. 33201321 10.1007/s00134-020-06312-y PMC7670292

[10] Long B Liang SY Lentz S. High flow nasal cannula for adult acute hypoxemic respiratory failure in the ED setting. Am J Emerg Med. 2021;49:352–9. 34246166 10.1016/j.ajem.2021.06.074 PMC8555976

[11] Yan H Qinghua L Mengyuan P. High flow nasal cannula therapy for obstructive sleep apnea in adults. Sleep Breath. 2022;26:783–91. 34383275 10.1007/s11325-021-02453-6

[12] Yu CC Huang CY Hua CC Wu H-P. High-flow nasal cannula compared with continuous positive airway pressure in the treatment of obstructive sleep apnea. Sleep Breath. 2022;26:549–58. 34145538 10.1007/s11325-021-02413-0

[13] Tsai FC Chen NL Gobindram A Singh PA Hsu PP Tan AKL. Efficacy of high flow nasal cannula as an alternative to continuous positive airway pressure therapy in surgical patients with suspected moderate to severe obstructive sleep apnea. Am J Otolaryngol. 2022;43:103295. 34922258 10.1016/j.amjoto.2021.103295

[14] Sowho MO Woods MJ Biselli P McGinley BM Buenaver LF Kirkness JP. Nasal insufflation treatment adherence in obstructive sleep apnea. Sleep Breath. 2015;19:351–7. 25015548 10.1007/s11325-014-1027-4

[15] Nakanishi N Suzuki Y Ishihara M. Effect of high-flow nasal cannula on sleep-disordered breathing and sleep quality in patients with acute stroke. Cureus. 2020;12:e9303. 32832300 10.7759/cureus.9303 PMC7437095

[16] Ho CH Chen CL Yu CC Yang Y-H Chen C-Y. High-flow nasal cannula ventilation therapy for obstructive sleep apnea in ischemic stroke patients requiring nasogastric tube feeding: a preliminary study. Sci Rep. 2020;10:8524. 32444630 10.1038/s41598-020-65335-z PMC7244586

[17] Sakaguchi Y Nozaki-Taguchi N Hasegawa M Ishibashi K Sato Y Isono S. Combination therapy of high-flow nasal cannula and upper-body elevation for postoperative sleep-disordered breathing: randomized crossover trial. Anesthesiology. 2022;137:15–27. 35471655 10.1097/ALN.0000000000004254

[18] Haba-Rubio J Andries D Rey V Michel P Tafti M Heinzer R. Effect of transnasal insufflation on sleep disordered breathing in acute stroke: a preliminary study. Sleep Breath. 2012;16:759–64. 21853283 10.1007/s11325-011-0572-3

[19] Spicuzza L Sambataro G Schisano M Ielo G Mancuso S Vancheri C. Nocturnal nasal high-flow oxygen therapy in elderly patients with concomitant chronic obstructive pulmonary disease and obstructive sleep apnea. Sleep Breath. 2023;27:1049–55. 36057738 10.1007/s11325-022-02702-2 PMC10227143

[20] Rotenberg BW Murariu D Pang KP. Trends in CPAP adherence over twenty years of data collection: a flattened curve. J Otolaryngol Head Neck Surg. 2016;45:43. 27542595 10.1186/s40463-016-0156-0 PMC4992257

[21] Weaver TE. Novel aspects of CPAP treatment and interventions to improve CPAP adherence. J Clin Med. 2019;8:2220. 31888148 10.3390/jcm8122220 PMC6947399

[22] Kwok KL Lau MY Leung SY Ng DK. Use of heated humidified high flow nasal cannula for obstructive sleep apnea in infants. Sleep Med. 2020;74:332–7. 32905994 10.1016/j.sleep.2020.08.005

[23] Du F Gu YH He YC Deng W-F Liu Z-Z. High-flow nasal cannula therapy for pediatric obstructive sleep apnea: a systematic review and meta-analysis. Eur Rev Med Pharmacol Sci. 2022;26:4583–91. 35856347 10.26355/eurrev_202207_29179

[24] Park S. High-flow nasal cannula for respiratory failure in adult patients. Acute Crit Care. 2021;36:275–85. 35263823 10.4266/acc.2021.01571 PMC8907461

[25] Karuga FF Kaczmarski P Białasiewicz P. REM-OSA as a tool to understand both the architecture of sleep and pathogenesis of sleep apnea-literature review. J Clin Med. 2023;12:5907. 37762848 10.3390/jcm12185907 PMC10531579

[26] Saletu B Oberndorfer S Anderer P. Efficiency of continuous positive airway pressure versus theophylline therapy in sleep apnea: comparative sleep laboratory studies on objective and subjective sleep and awakening quality. Neuropsychobiology. 1999;39:151–9. 10087460 10.1159/000026575

