# Supplementary material for: Effect of high-flow nasal cannula therapy on adults with obstructive sleep apnea: A meta-analysis
Source: Medicine (Baltimore). 2025 Nov 28;104(48):e45782. doi: 10.1097/MD.0000000000045782 (PMC12662469; doi:10.1097/MD.0000000000045782)
Supplement: Supplementary file 1 [file medi-104-e45782-s001.pdf]

**Supplementary Table S1 Search Strategy**

| Pubmed | |
|---|---|
| #1 | "high flow nasal"[Title/Abstract] OR "high-flow nasal"[Title/Abstract] OR "highflow nasal"[Title/Abstract] OR HFNC[Title/Abstract] OR HFNO[Title/Abstract] OR "high flow oxygen"[Title/Abstract] OR "long-term humidification"[Title/Abstract] OR "nasal high flow"[Title/Abstract] |
| #2 | "Sleep Apnea Syndromes" [Mesh] OR "Sleep Apnea, Obstructive" [Mesh] |
| #3 | OSA[Title/Abstract] OR OSAHS[Title/Abstract] OR SAHS[Title/Abstract] OR Sleep Hypopnea*[Title/Abstract] OR "Obstructive Sleep"[Title/Abstract] OR "sleep apnoea" [Title/Abstract] OR "nocturnal apnea" [Title/Abstract] OR "nocturnal apnoea" [Title/Abstract] OR sleep respiratory disorder*[Title/Abstract] OR sleep breathing disorder*[Title/Abstract] OR"sleep disordered breathing" [Title/Abstract] |
| #4 | #2 OR #3 |
| #5 | #1 AND #4 |

| Embase | |
|---|---|
| #1 | 'high flow nasal':kw,ti,ab OR 'high-flow nasal':kw,ti,ab OR 'highflow nasal':kw,ti,ab OR 'HFNC':kw,ti,ab OR 'HFNO':kw,ti,ab OR 'high flow oxygen':kw,ti,ab OR 'long-term humidification':kw,ti,ab OR 'nasal high flow':kw,ti,ab |
| #2 | 'sleep disordered breathing'/exp |
| #3 | OSA:kw,ti,ab OR OSAHS:kw,ti,ab OR SAHS:kw,ti,ab OR Sleep Hypopnea*:kw,ti,ab OR 'Obstructive Sleep':kw,ti,ab OR 'sleep apnoea':kw,ti,ab OR 'nocturnal apnea':kw,ti,ab OR 'nocturnal apnoea':kw,ti,ab OR sleep respiratory disorder*:kw,ti,ab OR sleep breathing disorder*:kw,ti,ab OR 'sleep disordered breathing':kw,ti,ab |
| #4 | #2 OR #3 |
| #5 | #1 AND #4 |

| Cochrane CENTRAL | |
|---|---|
| #1 | "high flow nasal" OR "high-flow nasal" OR "highflow nasal" OR HFNC OR HFNO OR "high flow oxygen" OR "long-term humidification" OR "nasal high flow" |
| #2 | MeSH descriptor: [Sleep Apnea Syndromes] explode all trees |
| #3 | MeSH descriptor: [Sleep Apnea, Obstructive] explode all trees |
| #4 | OSA OR OSAHS OR SAHS OR Sleep Hypopnea* OR "Obstructive Sleep" OR "sleep apnoea" OR "nocturnal apnea" OR "nocturnal apnoea" OR sleep respiratory disorder* OR sleep breathing disorder* OR"sleep disordered breathing" |
| #5 | #2 OR #3 OR #4 |
| #6 | #1 AND #5 |

**Supplementary Table S2 Quality Assessment Tool for Before-After (Pre-Post) Studies With No Control Group**

| Quality Assessment Tool for Before-After (Pre-Post) Studies With No Control Group | | | | | | | | | | | | |
|---|---|---|---|---|---|---|---|---|---|---|---|---|
| **Study** | **1** | **2** | **3** | **4** | **5** | **6** | **7** | **8** | **9** | **10** | **11** | **12** |
| Li 2024 | Y | Y | Y | NR | N | Y | Y | NR | NA | Y | NA | NA |
| Spicuzza 2022 | Y | Y | Y | NR | N | Y | Y | NR | NA | Y | NA | NA |
| Yuichi 2022 | Y | Y | Y | NR | N | Y | Y | N | NA | Y | NA | NA |
| Tsai 2022 | Y | Y | Y | NR | N | Y | Y | Y | NA | Y | NA | NA |
| Yan 2021 | Y | Y | Y | NR | N | Y | Y | NR | NA | Y | NA | NA |
| Yu 2021 | Y | Y | Y | NR | N | Y | Y | NR | NA | Y | NA | NA |
| Ho 2020 | Y | Y | Y | NR | N | Y | Y | NR | NA | Y | NA | NA |
| Nobuto 2020 | Y | Y | Y | NR | N | Y | Y | NR | NA | Y | NA | NA |
| Sowho 2015 | Y | N | Y | NR | N | Y | Y | NR | NA | Y | NA | NA |
| Haba-Rubio 2012 | Y | N | Y | NR | N | Y | Y | Y | NA | Y | NA | NA |
| Nilius 2010 | Y | Y | Y | NR | N | Y | Y | NR | NA | N | NA | NA |
| McGinley 2007 | Y | Y | Y | NR | N | Y | Y | NR | NA | Y | NA | NA |

**Abbreviations:** Y, yes; N, no; NA, not applicable; NR, not reported. (1) Objective clearly stated; (2) eligibility criteria described; (3) representative patient population; (4) all eligible participants enrolled in study; (5) sufficient sample size; (6) intervention described; (7) outcome measures specified; (8) outcome assessors blinded; (9) loss to follow-up; (10) statistical analysis of outcome measures before and after intervention; (11) interrupted time-series design; (12) individual data used for group-level effects.

**Supplementary Figure S1**

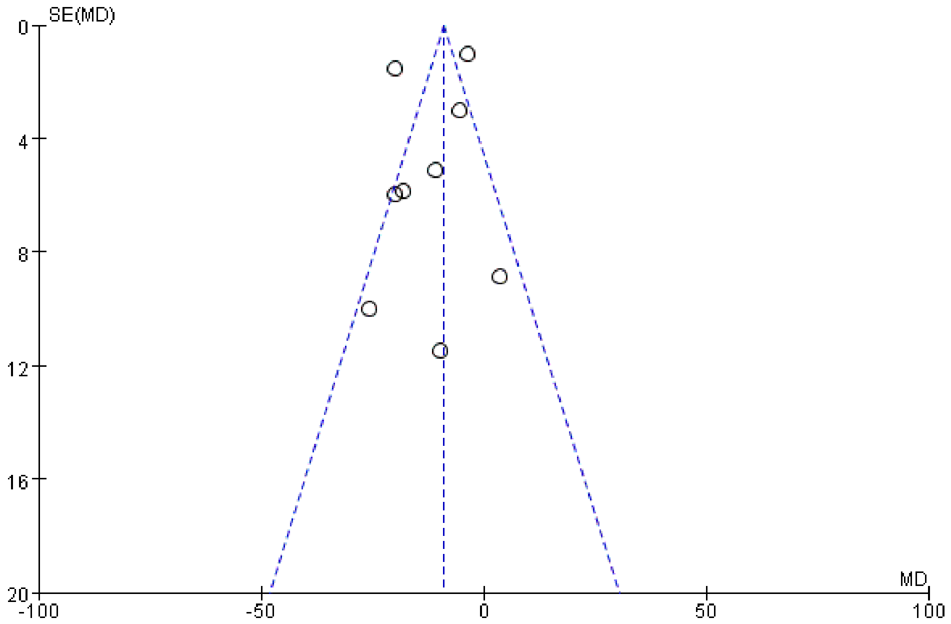


Figure S1: Publication bias analysis of AHI by funnel plot.

**Supplementary Figure S2**

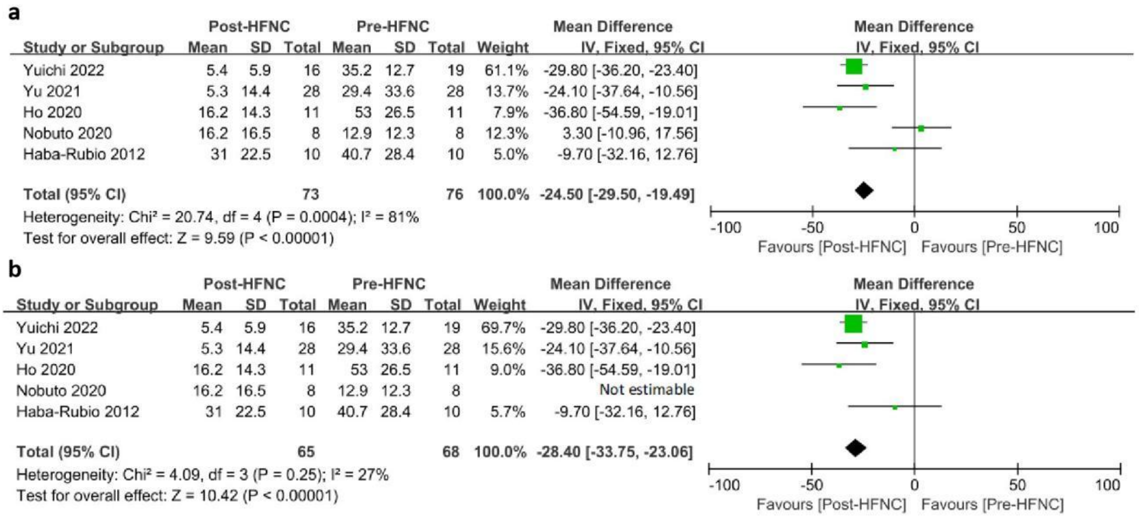


Figure S2 a: Forest plot of the effect of HFNC therapy on oxygen desaturation index. b: Forest plot of the effect of HFNC therapy on oxygen desaturation index (removing studies for heterogeneity).

**Supplementary Figure S3**

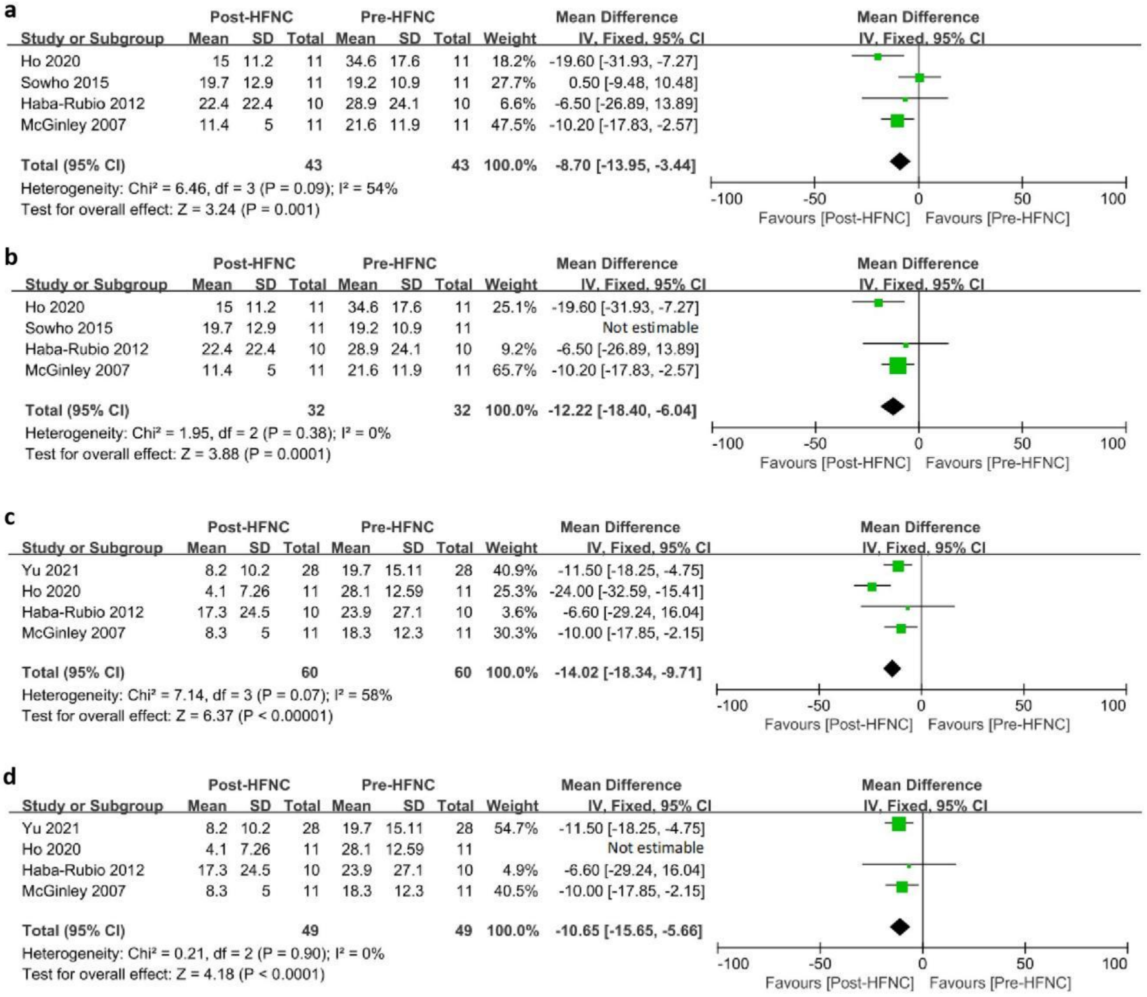


Figure S3 a: Forest plot of the effect of HFNC therapy on total arousal index. b: Forest plot of the effect of HFNC therapy on total arousal index (removing studies for heterogeneity). c: Forest plot of the effect of HFNC therapy on respiratory arousal index. d: Forest plot of the effect of HFNC therapy on respiratory arousal index (removing studies for heterogeneity).
